# Novel therapeutic strategies targeting HIV integrase

**DOI:** 10.1186/1741-7015-10-34

**Published:** 2012-04-12

**Authors:** Peter K Quashie, Richard D Sloan, Mark A Wainberg

**Affiliations:** 1 McGill University AIDS Centre, Lady Davis Institute, Montreal, Canada; 2 Division of Experimental Medicine, McGill University, Montreal, Canada; 3 Department of Microbiology and Immunology, McGill University, Montreal, Canada

**Keywords:** crystal structure, dolutegravir, HIV integrase, mutations, new drugs, raltegravir, resistance

## Abstract

Integration of the viral genome into host cell chromatin is a pivotal and unique step in the replication cycle of retroviruses, including HIV. Inhibiting HIV replication by specifically blocking the viral integrase enzyme that mediates this step is an obvious and attractive therapeutic strategy. After concerted efforts, the first viable integrase inhibitors were developed in the early 2000s, ultimately leading to the clinical licensure of the first integrase strand transfer inhibitor, raltegravir. Similarly structured compounds and derivative second generation integrase strand transfer inhibitors, such as elvitegravir and dolutegravir, are now in various stages of clinical development. Furthermore, other mechanisms aimed at the inhibition of viral integration are being explored in numerous preclinical studies, which include inhibition of 3' processing and chromatin targeting. The development of new clinically useful compounds will be aided by the characterization of the retroviral intasome crystal structure. This review considers the history of the clinical development of HIV integrase inhibitors, the development of antiviral drug resistance and the need for new antiviral compounds.

## Review

### Early integrase inhibitors

HIV integrase (IN) is pivotal in the viral replication cycle as it catalyzes the insertion of the reverse transcribed viral genome into host chromatin. Integrase catalyzes two distinct steps, 3' processing and strand transfer. During 3' processing, integrase excises a dinucleotide from the 3' terminus of viral cDNA. This 3'-processed viral DNA is then covalently linked to host DNA during strand transfer [1]. This unique process has always been considered a viable drug target, which several early studies attempted to exploit [2]. Early integrase inhibitors (INIs) included peptides [3,4], nucleotides [5] and DNA complexes [6] as well as small molecules derived either from natural products [5] or by rational drug design strategies [4,7]. Even though some of these compounds advanced into preclinical trials, further clinical development was always curtailed due to *in vivo *toxicity and/or non-specific off-target effects. More detailed reviews on the development of early INIs have been published [2,4,8].

For any inhibitor to be considered useful as an antiviral in combination therapy for HIV, selectivity (such as for IN) that is distinct from effects on other targets (such as RT and protease) needs to be proven. The 4-aryl-2,4-diketobutanoic acid inhibitors containing a distinct diketo acid moiety (DKA) were identified in 2000 by Merck investigators from a screen of 250,000 compounds, and for a time were the only biologically validated INIs [9]. Their antiviral activity in cell culture was mitigated by the development of resistance mutations in the IN protein, thereby confirming their mode of action [9]. These compounds, exemplified by L-731988 [10], were found to inhibit strand transfer with much higher potency (half-inhibitory concentration (IC50) = 80 nM) than 3' prime processing (6 μM) [9], and they were thus referred to as integrase strand transfer inhibitors (INSTIs). IN, like most nucleotidyltransferase enzymes, requires two divalent cations bound at the active site for activity; Mg^2+ ^is likely used *in vivo*, although Mn^2+ ^is used in some *in vitro *assays [11]. Most INSTIs that have been described, including DKA compounds, inhibit IN by chelation of bound cations in a dose-dependent manner [12]. The crystal structure [13] of IN bound to the prototype DKA, 1-(5-chloroindol-3-yl)-3-hydroxy-3-(2H-tetrazol-5-yl)-propenone (5-CITEP) [14] provided structural evidence for DKA-IN binding interactions. The compound termed 5-CITEP was found to bind in proximity to the evolutionarily conserved D64 D116 E152 motif of IN, also providing valuable structural confirmation of the IN active site [13]. Subsequent variations of DKAs based on the 5-CITEP backbone led to increased potency, specificity, tolerability and bioavailability. This, in turn, led to the first clinically tested INI (S-1360). Despite an initially good pharmacological and pharmacokinetic profile in animal models, S-1360 in initial human trials was found to be rapidly cleared through glucuronidation [15] and its development was curtailed.

### First generation clinically approved integrase inhibitors

#### Raltegravir

**Figure 1:**
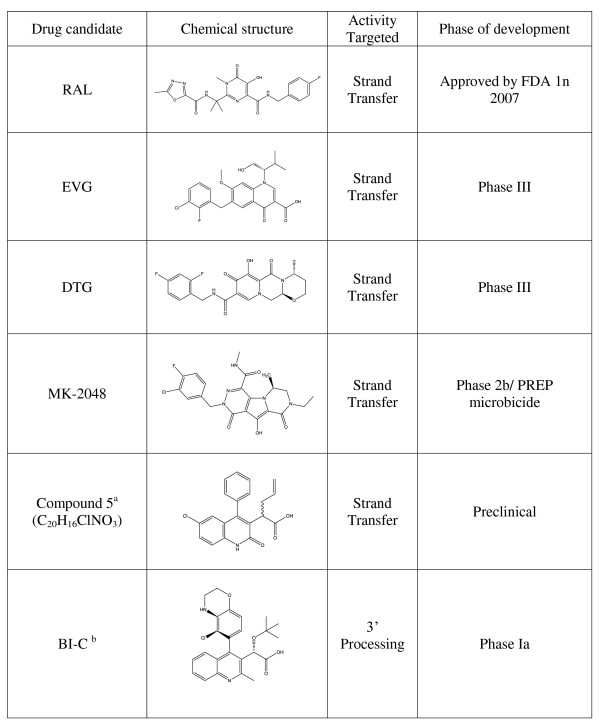
**Clinically relevant or promising HIV integrase inhibitor compound structures**. ^a^Based on nomenclature from [109]; ^b^structure of BI-C, a precursor to BI 224436.

Optimization of lead compounds including L-31988 and L-870812 by Merck pharmaceuticals led to the development of raltegravir (RAL; Isentress, MK-0518) (Figure 1), which in 2007 became the first (and currently only) INI approved for treatment in both antiretroviral (ARV) naïve and treatment-experienced patients [16]. RAL was shown in multiple trials, such as BENCHMRK, to achieve efficient viral load suppression in ARV-experienced patients when included in an optimized background ARV regimen [17]. In the BENCHMRK trials, 57% of patients achieved plasma levels of HIV-1 RNA < 50 copies/mL after 97 weeks of therapy, whereas only 26% of the placebo group, treated with optimized background regimen (OBR) drugs, achieved viral suppression. The efficacy of RAL relative to other ARVs has been modeled in cell culture and has been shown to be owing to the activity of INIs at later stages in the viral replication cycle than either viral entry or reverse transcription inhibitors: they are therefore able to inhibit replication in a larger proportion of productively infected cells [18]. In another study of patients with multidrug-resistant viruses with a median ARV treatment experience of 9 years, a RAL-containing regimen yielded higher viral load suppression than a regimen containing placebo when combined with OBR [19]. RAL has a favorable toxicity profile and does not appear to have a high propensity for clinically relevant drug-drug interactions [11], except for minor induction of the glucuronidation enzyme UGT1A1 responsible for RAL elimination [20]. Interactions with drugs such as rifampin may lead to modest decreases in RAL half-life and blood concentration after 12 hours (C12hr). Predictably, other UGT1A1 inhibitors, such as atazanavir, have been shown to exert a modest but not clinically relevant extension of C12hr levels for RAL. RAL has been shown to have high bioavailability and is dosed twice daily at 400 mg/mL due to its C12hr of 142 nM [21]. Studies to simplify RAL dosage to 800 mg once daily, boosted or unboosted by the UGT1A1 inhibitor atazanavir, have not yielded significant promise [22-24].

Despite the high effectiveness of RAL for first-line and salvage therapy, resistance mutations can reduce the susceptibility of the virus to INIs. The occurrence of single point mutations that confer high-level resistance (fold change (FC) > 5) to INIs have shown that RAL has a modest genetic barrier to resistance development. To date, three major resistance pathways involving non-polymorphic residues have been extensively described and characterized for RAL; E92QV/N155H, T97A/Y143CHR and G140CS/Q148HKR [25,26]. Although these three pathways have been shown to arise separately, some recent reports suggest that they may be linked. The G140S/C and E92Q/V mutations by themselves impart greater than five- to ten-fold resistance to RAL [27], but usually appear only after the N155H and Q148HKR mutations [28], leading to a FC > 100 for the combined mutations. In addition to these major resistance mutations, several polymorphic and non-polymorphic residues have been identified that impart a greater than five-fold resistance to RAL. Some of these, such as T66I/L, have been shown to act synergistically with pre-existing major resistance mutations [29]. All major INI resistance mutations have a major impact on both IN activity and viral replication capacity [30]. The result is a swift reversion to wild-type virus in patients soon after therapy with INIs is withdrawn [31].

It has been suggested that patients without a history of nucleoside reverse transcriptase inhibitor (NRTI)-associated resistance may have an increased barrier for the occurrence of resistance to RAL compared with patients with resistance to non-NRTIs, such as nevirapine and efavirenz (EFV) [32]. Most reported virologic failures due to RAL-resistance mutations have occurred in patients harboring NRTI-resistant viruses or in patients at increased risk of virologic failure [33]. This was highlighted in the SWITCHMRK1 and 2 phase III trials in patients undergoing salvage therapy with lopinavir, a protease inhibitor, and who switched from lopinavir (LPV) to RAL, despite having undetectable viremia. The results showed that 84.4% of those who switched to RAL (n = 353) maintained undetectable levels of viremia compared to > 90% in the treatment group who did not switch (n = 354). Thus, this study failed to establish non-inferiority of RAL to LPV in the treatment of ARV-experienced individuals with HIV with undetectable viremia [33]. Of the 11 patients who experienced virologic failure with HIV-1 RNA levels > 400 copies/mL, eight harbored RAL-resistance mutations [34].

#### Elvitegravir

Elvitegravir (EVG) (GS-9137) is not a DKA but a monoketo acid resulting from early modification of the DKA motif by the Japan Tobacco Company (Figure 1) [35]. This work resulted in a group of 4-quinolone-3-glyoxylic acids, all of which had a single pair of coplanar ketone and carboxylic groups and retained high specificity for and efficacy against the strand transfer reaction similar to DKA compounds [36]. EVG, now being developed by Gilead Sciences, has been shown to have an *in vitro *IC_50 _of 7 nM against IN and an antiviral (90% effective concentration (EC_90_) of 1.7 nM when assayed in the presence of normal human serum [37]. EVG displayed approximately 30% bioavailability in dogs and rats with maximal plasma concentrations being achieved 0.5 to 1 hour post dose [37]. In clinical trials, EVG was found to be well tolerated and efficacious [38]. Pharmacokinetic boosting with ritonavir (RTV) was found to result in improved dose-dependency [39].

The cytochrome p450 enzyme CYP3A4/5 is the primary metabolizing enzyme for EVG, followed by glucuronidation by UGT1A1/3 [39]. Thus, the bioavailability and clearance of EVG was found to be favored when EVG was dosed in combination with CYP3A4/5 inhibitors [11,39,40]. The CYP3A4/5 inhibitor, RTV, was found to cause an approximate 20-fold increase in the area under the curve and to extend elimination half-life from three to ten hours [41]. In a phase II trial of ARV-naïve patients (n = 48) starting initial therapy on an OBR of tenofovir/emtricitabine (TDF/FTC), the co-administration of EVG with a novel pharmacokinetic booster, cobicistat, in a single tablet formulation resulted in undetectable viremia in 90% of patients after 48 weeks compared with 83% of patients who received TDF/FTC/EFV [42]. In a Phase IIb study, RTV-boosted EVG was non-inferior to the RTV-boosted protease inhibitors darunavir and tipranavir when used in combination with other drugs [43].

A major drawback to the clinical uptake of EVG, despite it being a once-daily drug, may be that it shares a moderate genetic barrier to INI resistance with RAL and that extensive cross-resistance exists between the two compounds. The RAL signature mutations N155H, Q148H/R/K and G140A/C/S, as well as associated accessory mutations, were selected by EVG in culture [44] and in patients [33,45]. This precludes the use of EVG to treat most RAL-resistant viruses. The only major RAL-associated mutations not selected by EVG wereY143C/R/H and subsequent studies showed that viruses containing Y143C/R/H remained susceptible to EVG [46]. In addition to RAL-associated resistance mutations, EVG selected for other mutational pathways. T66I did not confer high-level resistance to RAL [44], but conferred a > 10-fold resistance to EVG, while a T66R mutation conferred > 10-fold resistance to RAL and > 80-fold resistance to EVG [47,48]. The T66I mutation is associated with a series of accessory mutations, including F121Y, S153Y and R263K; the latter two have not been associated with RAL-resistance [49]. AF121Y mutation has been selected with RAL and confers high-level resistance to this compound, but has not yet been identified in the clinic [47]. Other clinically selected EVG mutations are S147G, which confers > eight-fold resistance to EVG but does not affect susceptibility to RAL [47]. Other *in vitro *EVG selections resulted in several high resistance mutations that have yet to be clinically validated, such as P145S, Q146P and V151A/L [47]. The V151L mutation confers an approximate eight-fold cross-resistance to RAL and has been identified in a single patient treated with RAL [50].

### Second generation integrase inhibitors

#### MK-2048

The discovery of a low-to-moderate genetic barrier of resistance with first generation INIs led to efforts to produce second generation INSTIs with activity against RAL-resistant viruses. Optimization of tricyclic 10-hydroxy-7,8-dihydropyrazinopyrrolopyrazine-1,9-dione compounds led to the development of MK-2048 [51] (Figure 1), which demonstrates a EC_95 _< 50 nM when assayed in 50% human serum and possesses a favorable pharmokinetic profile in dogs and rats [52]. MK-2048 was subsequently shown in tissue culture and biochemical assays to be effective against RAL- and EVG- resistant viruses [51-55], with only slightly diminished effectiveness against viruses containing at least two of the following mutations: E138K, G140S and Q148R [51-55]. Selection studies in culture with MK-2048 did not select for previously recognized mutations but instead selected a novel substitution at position G118R that, in concert with E138K, conferred approximately eight-fold resistance to MK-2048 [56]. Despite its favorable resistance profile, MK-2048 has a poor pharmacokinetic profile and its clinical development has been arrested. However, it has potential as a candidate microbicide for prevention of HIV infection [57]. It continues to be studied as a prototype second generation INI and has also recently shown effectiveness in the treatment of human T-lymphotropic virus type 1 in culture without causing significant toxicity in target cells [58].

#### Dolutegravir

Dolutegravir (DTG) (S/GSK 1349572) is currently in phase III clinical trials (for structure, see Figure 1). It was discovered at Shionogi Pharmaceuticals in Japan and is now being developed by a Shiniogi-ViiV Healthcare-GlaxoSmithKline joint venture [59,60]. DTG is a promising HIV INI candidate that specifically inhibits the strand transfer reaction with recombinant purified integrase [60]. Inhibition of the integrase strand transfer reaction by DTG has been confirmed in studies with live virus, which demonstrated an accumulation of 2-long terminal repeat (2-LTR) circles in treated cells at DTG concentrations < 1,000-fold of those that caused cell toxicity [61,62]. DTG also demonstrated efficacy against most viral clones resistant to RAL and EVG and against clinical isolates of HIV-1 and HIV-2, although some viruses containing E138K, G140S or R148H mutations possessed diminished susceptibility to DTG [60,63-65]. Double mutants containing combinations of E138K, G140S and R148H had a FC > 10 for DTG, but this was favorable when compared to RAL, which yielded a FC of > 330 and > 140, respectively. *In vitro *combination antiviral studies showed that DTG did not increase toxicity when used in combination, but had a synergistic effect with each of EFV, nevirapine, stavudine, abacavir, LPV, amprenavir and enfuvirtide as well as an additive effect in combination with maraviroc. The hepatitis B virus drug adefovir and the hepatitis C virus drug ribavirin had no effect on the efficacy of DTG [65], allowing for its potential use in treating co-infections.

The pharmacokinetic profile of DTG allows once-daily dosing without pharmacokinetic boosting. This is based on a long unboosted half-life (13 to 15 hours) with trough levels of DTG being much higher than the *in vitro *IC_90 _[66]. The side-effects of DTG in volunteers with HIV infection were similar to those of placebo in phase I clinical trials [66].

Phase IIa randomized double blind trials provided vital evidence of the anti-HIV effect and potency of DTG [67,68]. Notably, 35 ARV-experienced INI-naïve patients, who were not receiving therapy, and whose plasma HIV-1 RNA levels ranged from 3.85 to 5.54 log copies/mL, received once-daily doses of 2 mg, 10 mg or 50 mg DTG or placebo for 10 days. More than 90% of patients who received DTG, irrespective of dose, had a decline in viral load to < 400 copies/mL while 70% of patients in the 50 mg arm achieved undetectable viremia. In contrast, the placebo group showed an average increase in viremia. No serious adverse effects were reported in this trial, with headaches and pharyngolaryngeal pain being the most commonly reported consequence [67].

In the SPRING-1 double blind dose-ranging phase II trials, 205 ARV-naïve patients with HIV, with CD4^+ ^cells > 200 cells/mm^3 ^and HIV-1 RNA > 1,000 copies/mL, were treated once daily with DTG (n = 155) at10 mg, 25 mg or 50 mg doses or 600 mg EFV (n = 50) combined with background therapy of TDF/FTC or abacavir/3TC [69]. More than 90% of all participants in the DTG arm had undetectable viremia after 24 weeks of treatment, establishing the non-inferiority of DTG to EFV in an NRTI or non-NRTI background and also showing that DTG was at least as safe as EFV.

No primary INI resistance mutations have yet been reported for DTG either in culture or in the clinic. Tissue culture selection studies over 112 weeks identified, in order of appearance, viruses harboring T124S/S153F, T124A/S153Y, L101I/T124A/S153F and S153Y by week 84. Although these mutations persisted throughout serial passaging, they did not confer high-level resistance to DTG [65]. Position 124 of IN is modestly polymorphic and S153F/Y had previously been described in EVG selection studies [70]. Despite an apparently high genetic barrier for resistance, selection, recent tissue culture and biochemical studies report that a R263K mutation in IN may confer modest resistance to DTG [71].

It has been suggested that DTG enjoys a high barrier for resistance due to a tighter binding of DTG to IN compared to RAL and EVG [72]. Assays also showed that DTG exhibited tighter binding and had a longer dissociative half-life from IN than either RAL or EVG [73].

In this model, a direct relationship existed between the half-life of binding and the inhibitory potential of INIs when the binding half-life (t_1/2_) was below 4 hours. A > 3 FC in regard to drug resistance, relative to the wild-type, was observed when the t_1/2 _dropped below 1 hour [72]. In assays with wild-type enzymes, the t_1/2 _of DTG, RAL and EVG were 71, 8.8 and 2.7 hours, respectively. The fact that RAL and EVG have a shorter t_1/2 _than DTG suggests that resistance mutations that affect binding of RAL and EVG might also be more likely to compromise antiviral potency. As an example, the Y143CHR mutations have been shown to compromise interactions between IN and RAL but not those between IN and DTG or between IN and EVG [74]. This is further supported by data on mutations that have been shown to significantly reduce t_1/2_, E92Q/N155H, E138K/Q148R and G140S/Q148R, and significantly reduce antiviral potency [72]. This hypothesis had been previously suggested for MK-2048, which also has a relatively high barrier for resistance, as it also has a slower off-rate (t_1/2 _= 32 hours) for IN compared to RAL (t_1/2 _≥ 7.3 hours) [73].

The use of DTG in INI-salvage therapy is being investigated in an ongoing study called VIKING. The latter is a phase II single arm study investigating the feasibility of replacing RAL with DTG in patients experiencing failure due to RAL-resistant viruses [59]. Participants (n = 27) were switched from their previous RAL-containing regimens to receive DTG 50 mg once daily for 10 days and were then prescribed other active drugs over a period of 23 weeks. Eighteen of the study participants had INI- resistant viruses belonging to the Y143, Q148 and N155 pathways prior to initiation of the study. After 10 days of DTG monotherapy, all participants harboring viruses in the Y143 and N155 pathways attained a mean HIV-1 RNA decrease of approximately 1.8 log copies/mL compared with approximately 0.7 log copies/mL for viruses harboring G140S/Q148HRK double mutations. None of the viruses harboring Q148HRK plus two or more additional mutations experienced a decrease of ≥ 0.7 log copies/mL, indicating a degree of resistance on the part of Q148HRK viruses to DTG. This trial nonetheless provided proof-of-principle for the use of DTG in RAL-experienced patients infected by subtype-B viruses harboring position Y143 and N155 mutations.

In order to model the effects of DTG in RAL-experienced patients, several serial passaging studies have been carried out and shown that the presence of the N155H and Y143CHR resistance did not lead to development of additional resistance mutations under DTG pressure nor to a substantial decrease in DTG susceptibility [62,64]. In contrast, the presence of Q148HRK mutations did lead to further mutations and > 100 FC for DTG susceptibility relative to wild-type in subtype B viruses [63,65]. Interestingly, Q148HRK mutations did not affect susceptibility to DTG in HIV-1 subtype C and HIV-2 isolates [65,75,76]. An ongoing trial termed SPRING-2 will evaluate the use of once-daily DTG versus twice-daily RAL in treatment-naïve patients. A Phase III trial termed SAILING will compare once-daily versus twice-daily DTG in ARV-experienced INI-naïve participants with HIV [77].

#### S/GSK-1265744

Another second generation INSTI called S/GSK-1265744, which is a back-up drug to DTG, has been tested in double blind randomized placebo-controlled trials and has shown promising short-term efficacy, an excellent pharmacokinetic profile and good tolerability in patients with HIV [78]. Its future development is uncertain, however, given the positive state of development and promise of DTG.

### Advances aiding integrase inhibitor discovery

#### Crystallization of full-length integrase

Due to the low solubility of HIV-1 IN [79,80], elucidation of the full-length IN structure has never been accomplished. The first IN partial-structure was published in 1994 [81]; however, despite the insights afforded by this and subsequent structures, including the first partial IN structure complexed with an inhibitor [13], none of these structures gave a proper depiction of inhibitor drug interactions, IN-DNA interactions or functional IN quaternary structures. Co-crystal structures of integrase from the lentivirus Maedi-Visna with human LEDGF [82] suggested that the functional IN protein might be tetrameric, consisting of a dimer of dimers, and this further showed the necessity of obtaining full-length crystal structure for proper elucidation of IN structure, function and inhibition. In 2010, the full-length structures of IN from the prototype foamy virus (PFV) in complex with LTR mimetics were published [83]. This paper provided the first glimpse into interactions between IN and viral DNA and also established the binding mode of the INSTIs RAL and EVG. A follow-up publication [84] provided excellent structural explanations for the impact of mutations at positions 92, 140, 148 and 155 on RAL and EVG susceptibility. Thus, despite the fact that PFV is a spumavirus, only having significant sequence identity with HIV-1 IN in the catalytic core domain (CCD) domain, PFV IN structures could guide construction of reliable homology models of HIV-1 IN with accurate prediction of interactions between IN and INSTI [85]. Later crystallization efforts by the same group yielded IN-DNA strand transfer complexes in the presence and absence of inhibitors [86], again providing new structural data, a better understanding of the strand transfer process and information on new INI discovery initiatives. Co-crystallization studies have attributed the observed efficacy of DTG against RAL- and EVG-resistant viruses to the flexibility of DTG and its ability to bind to IN, even in the presence of major INI resistance mutations [74]. It remains to be seen whether PFV structures can aid in the elucidation of non-catalytic site INIs, given major differences that may exist distal from the IN active site. For instance, PFV integrase does not interact with LEDGF [87]; as such, models based on PFV may not be able to help in the design of IN-LEDGF inhibitors. Further insights into integration based on PFV structures are discussed in other reviews [88,89].

#### Quantitative structure-property and -activity relationships

The recent elucidation of the full-length PFV intasome and strand transfer complexes have allowed for the generation of homology models of HIV-IN that can be used to 'train' and score drug prototypes. There are multiple quantitative structure-property relationships (QSPR) and quantitative structure-activity relationships (QSAR) protocols and programs. Some of these require advanced programming and mathematical skills, but several stand-alone and online programs offer semi-automated drug docking and scoring capabilities with moderate to high accuracy. The main aim of these approaches is to allow *in silico *validation and testing of prototype molecules in order to lower the costs associated with large-scale synthesis of non-validated compounds [90]. Typical QSPR and QSAR protocols use a given set of conditions that train and/or test the structures and a set of validatory parameters that are then used to score the data. Structures can then be selected for subsequent synthesis and experimental validation [91]. Typical input takes into account the physicochemical properties of individual moieties on the compound, bond-length, flexibility, lipophilicity and/or hydrophilicity, information on the target and three-dimensional binding space. This can generate theoretical estimations of IC_50_, binding affinity, bioavailability, hepatic clearance and other parameters. Recent work has used a molecular dynamics approach to accurately predict potency of INSTIs based on models derived from the PFV structure [92]. A summary of computer-based approaches for design of novel INIs that target 3' processing, IN multimerization, strand transfer complex assembly and IN-host protein interactions has recently been published [93]. Despite these advances, it is difficult to accurately model drug toxicity, bioavailability and safety prior to the synthesis and study of novel compounds.

### Next-generation strand transfer inhibitors in preclinical development

The design of MK-0536 by Merck & Co., Inc. was based on QSPR and QSAR that took into account the optimum minimum structure necessary for activity and generated a set of potential structures that could be synthesized and screened. MK-0536 has shown low hepatocyte clearance values [94] and generally good inhibition of wild-type IN and RAL-resistant IN [94,95] but its current level of clinical development is unclear. Other classes of compounds that block strand transfer with high specificity at sub-nanomolar EC_50_s and low toxicities are catechol-based [96], pyrimidone-based [97-100], dihydroxypyrido-pyrazine-1,6-diones [101] and quinolones [102,103].

#### Inhibiting integrase-host factor interactions

The understanding that IN takes part in a number of interactions with host proteins and their post-translational modifications has led to the targeting of some of these processes (reviewed in [104,105]). Separate studies have shown that sumoylation [106] and acetylation [107] of IN occurs *in vivo*, leading to increased activity, but there are currently no inhibitors of these reactions that can specifically target IN modification without affecting other cellular proteins [108]. The observation that integration can be inhibited in *ex vivo *HIV-infected CD4^+ ^T-cells of elite controllers [108] has not yet led to the identification of a responsible cellular factor. Currently, the most promising inhibitors targeting IN-host interactions disrupt the interaction between IN and LEDGF/p75; the latter is a host protein that has been shown to be essential for tethering the IN pre-integration complex to host chromatin and also for the recruitment of other cellular factors to the pre-integration complex, thereby facilitating effective integration [109,110]. Inhibition of the LEDGF/p75-IN interaction can seriously inhibit viral replication [109,111]. This is supported by the recent finding that polymorphisms in the PSIP-1 gene that codes for LEDGF/p75 can affect rates of HIV disease progression [112].

#### Allosteric inhibitors

##### LEDGINS

LEDGINS (Figure 1) were designed as specific small molecular inhibitors of the LEDGF/p75 interaction. Optimized structures within the group of 2-(quinolin-3-yl)acetic acid derivatives co-crystallized with LEDGF/p75-IN were shown to inhibit the LEDGF/p75-IN interaction at submicromolar concentrations and to inhibit strand transfer activity of IN, even in the absence of LEDGF/p75 [109,111]. Peptides mimicking the IN binding domain of LEDGF/p75 exhibit potent inhibition of IN [113,114].

##### BI 224436

BI 224436 is a novel INI with a distinct mode of action from more established INSTIs. It is a non-catalytic site integrase inhibitor that, like the LEDGINs described above, interferes with the interaction between IN and the chromatin targeting the LEDGF/p75 protein, yielding low nanomolar inhibition of 3' processing and viral replication [115]. It is not yet clear why these two sets of allosteric inhibitors, binding in the same pocket, should specifically inhibit different IN activities. The profile of BI 224436 appears favorable and it also appears to be specific, since it did not exhibit reduced activity against any INSTI-resistant IN enzymes [115]. This compound has now entered phase Ia clinical trials to evaluate dosing and safety in healthy individuals. Initial reports indicate high bioavailability with good tolerability at single doses ranging up to 200 mg. BI 224436 also exhibited good dose-proportional pharmacokinetics when given as a single dose of 100 mg, and plasma levels appeared adequate to achieve a therapeutic effect [116]. There have been a number of recent in-depth reviews on the subject of LEDGF/p75 targeted INIs [111,114,117].

#### Dual reverse transcriptase and integrase inhibitors

The structural and functional similarities between HIV-1 IN and the RNAse-H domain of HIV-1 RT suggest the possibility of specific yet dual targeting inhibitors of both processes. Some early compounds that have been found to target both enzymes are DKAs [118,119]. This hybrid class has been comprehensively reviewed elsewhere [120].

#### HIV diversity and integrase inhibitors

Recent reports indicate that subtype differences may exist with regard to the development of resistance to IN inhibitors, a phenomenon that also exists with RT inhibitors [27,121,122]. Despite the fact that HIV-1 subtype B and C wild-type IN enzymes are similarly susceptible to clinically validated INIs [61], the presence of resistance mutations may differentially affect susceptibility to specific INSTIs [27]. Recent reports suggest that the G118R mutation, which was previously reported to confer slight resistance to MK-2048, imparts a 25-fold resistance to RAL when present together with the polymorphic mutation L74M in CRF-AG cloned patient isolates [123]. Additionally, it is well documented that the INI Q148RHK resistance mutations, which affect susceptibility to DTG in HIV-1 subtype B, may not affect the susceptibility of either HIV-1 subtype C or HIV-2 enzymes to DTG [72].

## Conclusions

The development of INSTIs has resulted in a new drug class in the anti-HIV armamentarium. New compounds are being developed that possess improved resistance profiles and pharmacokinetics. It therefore seems likely that INSTIs will be a future stalwart of antiretroviral therapy. These advances have been accompanied by improved understanding of IN function that, in turn, is leading to the identification of new molecules that can block IN function through novel mechanisms.

## Abbreviations

ARV: antiretroviral; DKA: diketo acid moiety; DNA: deoxyribonucleic acid; DTG: dolutegravir; EFV: efavirenz; EVG: elvitegravir; FC: fold change; HIV: human immunodeficiency virus; IN: integrase; INI: integrase inhibitor; INSTI: integrase strand transfer inhibitor; LEDGF: lens epithelium derived growth factor; LPV: lopinavir; LTR: long terminal repeats; NRTI: nucleoside reverse transcriptase inhibitor; OBR: optimized background regimen; PFV: prototype foamy virus; QSAR: quantitative structure-activity relationships; QSPR: quantitative structure-property relationships; RAL: raltegravir; RNA: ribonucleic acid; RT: reverse transcriptase; RTV: ritonavir; TDF/FTC: tenofovir/emtricitabine.

## Competing interests

The authors declare that they have no competing interests.

## Authors' contributions

PKQ, RDS and MAW wrote and edited the manuscript. All authors read and approved the final version of this manuscript.

## Pre-publication history

The pre-publication history for this paper can be accessed here:

http://www.biomedcentral.com/1741-7015/10/34/prepub
